# Potential therapeutic implications of new insights into respiratory syncytial virus disease

**DOI:** 10.1186/rr184

**Published:** 2002-06-24

**Authors:** Peter JM Openshaw

**Affiliations:** 1 Department of Respiratory Medicine (St Mary's), National Heart and Lung Division, Faculty of Medicine, Imperial College of Science, Technology and Medicine, London, UK

**Keywords:** antiviral agents, asthma, bronchiolitis, respiratory syncytial virus

## Abstract

Viral bronchiolitis is the most common cause of hospitalization in infants under 6 months of age, and 70% of all cases of bronchiolitis are caused by respiratory syncytial virus (RSV). Early RSV infection is associated with respiratory problems such as asthma and wheezing later in life. RSV infection is usually spread by contaminated secretions and infects the upper then lower respiratory tracts. Infected cells release proinflammatory cytokines and chemokines, including IL-1, tumor necrosis factor-α, IL-6, and IL-8. These activate other cells and recruit inflammatory cells, including macrophages, neutrophils, eosinophils, and T lymphocytes, into the airway wall and surrounding tissues. The pattern of cytokine production by T lymphocytes can be biased toward 'T-helper-1' or 'T-helper-2' cytokines, depending on the local immunologic environment, infection history, and host genetics. T-helper-1 responses are generally efficient in antiviral defense, but young infants have an inherent bias toward T-helper-2 responses. The ideal intervention for RSV infection would be preventive, but the options are currently limited. Vaccines based on protein subunits, live attenuated strains of RSV, DNA vaccines, and synthetic peptides are being developed; passive antibody therapy is at present impractical in otherwise healthy children. Effective vaccines for use in neonates continue to be elusive but simply delaying infection beyond the first 6 months of life might reduce the delayed morbidity associated with infantile disease.

## Introduction

The World Health Organization estimates that approximately 14 million people die each year from infections that are transmitted via the respiratory tract, most of which occur in childhood. Viral infections of the respiratory tract are particularly serious during infancy, and viral bronchiolitis is the most common cause of infantile hospitalization in the developed world [1]. It has been estimated to cause 91,000 admissions per year in the USA, with associated hospitalization costs of $300,000,000 per year. Respiratory syncytial virus (RSV) accounts for approximately 70% of all cases of viral bronchiolitis [2].

RSV bronchiolitis usually affects children under 1 year old, with a peak incidence at age 2–4 months [3]. During this period of development, the lungs are growing rapidly and undergoing alveolar septation. Bronchiolitis may cause severe insult to the lungs during this critical period, and might cause long-term effects by delaying or preventing normal postnatal pulmonary changes. This could result in smaller lungs in later life that are more susceptible to disease. Alternatively, neonatal infection may cause long-lasting changes in host immunity [4].

**Figure 1:**
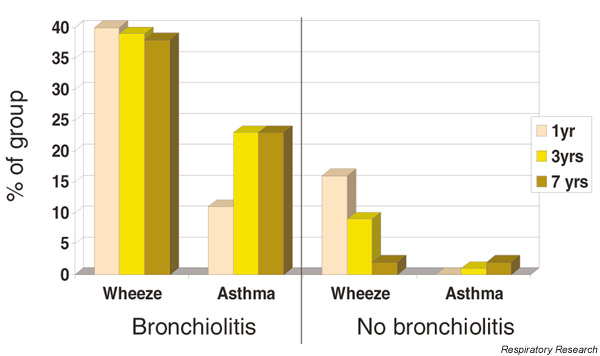
Association between bronchiolitis during infancy and wheezing or asthma in childhood. Infants who experience RSV bronchiolitis are more likely to develop wheezing and asthma at 1, 3, and 7 years of age than are matched controls who did not experience bronchiolitis as infants [5].

A recent study [5] showed that infants who experience RSV bronchiolitis are more likely to develop wheezing and asthma later in life (Fig. 1). This paper reviews the basic mechanisms of RSV infection, with particular reference to links between early RSV infection and the development of later respiratory symptoms and disease.

## Mechanism of respiratory syncytial virus infection

RSV belongs to the paramyxovirus family. It is transmitted by respiratory secretions and by direct contact with contaminated surfaces or materials. The most common way in which RSV is spread is by direct hand-to-hand contact. Infection occurs when the virus replicates in the tissue. Epithelial cells are the main target cells for respiratory viruses, but alveolar macrophages can also be infected.

Infection begins by binding to specific receptors on the surface of the host cell, followed by internalization and uncoating. The viral RNA core is then released into the cytoplasm of the host cell, where it replicates and is translated by the host cell machinery into viral particles. Viral assembly occurs in the cytoplasm and at the cell surface, after which virions are released from the cell. Released virions then infect other respiratory epithelial cells.

RSV was originally thought to be restricted to the respiratory tract, but recent studies have demonstrated viral RNA in peripheral blood cells during acute infection. It has been suggested that peripheral virus is not viable but data suggest it may be able to replicate [6]. In cattle, RSV appears able to persist in local B lymphocytes [7].

## Immune response to respiratory syncytial virus infection

Infected epithelial cells and alveolar macrophages activate the immune system's defenses. The cells release chemokines, proinflammatory cytokines, and mediators that include IL-1, tumor necrosis factor (TNF)-α, IL-6, IL-8, macrophage inflammatory protein (MIP)-1α and RANTES (regulated on activation, normal T cell expressed and secreted). Elevated levels of IL-6, IL-8, TNF-α [8], and IL-11 [9] have been found in nasal lavage fluid of children with acute upper respiratory infections. These cytokines and chemokines contribute to airway inflammation and bronchial hyperresponsiveness, as well as to mucus hypersecretion and sneezing.

Production of multiple cytokines and chemokines induces ingress into the airway of diverse inflammatory cells and their activation. IL-8 secretion causes influx of neutrophils, which are present in nasal secretions of children with rhinovirus upper respiratory infections [10] and in bronchial secretions of children with RSV infection [11]. RANTES and MIP-1α are potent chemokines for eosinophils. Increased levels of RANTES and MIP-1α have been found in the nasal aspirates of children with virus-induced asthma exacerbations [12] and in lower airway secretions of infants with RSV bronchiolitis who are given ventilatory support [13]. The viral inflammatory response includes activation of cytotoxic T cells. Virus-specific cellular cytotoxic activity has been demonstrated in infants with acute RSV infection [14]. Once in the airway, recruited cells prolong inflammation by releasing further cytokines and chemokines that attract and upregulate other inflammatory cells.

T-helper (Th) cells are found in the airways of individuals with RSV infection [15]. T cells produce proinflammatory mediators that include type 1 cytokines or type 2 cytokines. Th1 cells secrete IL-2, IFN-γ, and lymphotoxin, whereas Th2 cells secrete IL-4, -5, -6, and -10. IL-4 and IL-5 promote IgE production and eosinophilia [16]. These types of T-helper lymphocytes are selectively promoted, depending on the cytokine environment that is present during priming (largely determined by innate immune responses, site, concentration of antigen, and infection history). The balance between Th1 and Th2 responses is important in coordinating the protective and immunopathogenic responses to viral infection [17,18]. The immune system undergoes rapid development prenatally and during the first months of postnatal life. Infants show a greater propensity to develop Th2 responses than do older children.

### Antibody responses

In the newborn, passively acquired maternal immunoglobulin appears to protect against infection. This may be the reason why children under 8 weeks of age are rarely infected with RSV. Maternal antibody concentration decreases during the first 6 months of life, leaving infants between the ages of 2 and 4 months unprotected against infection. RSV bronchiolitis is most common in children younger than 6 months old. More than two decades ago, studies showed that infants with the highest titers of transplacentally acquired RSV-specific IgG developed less severe RSV pneumonia and had fewer infections than did infants with lower titers [19]. Other studies suggest that passively acquired maternal antibody suppresses the development of the infant's own immune response [20,21].

Local mucosal B cells can produce IgA, the predominant immunoglobulin in respiratory secretions. IgE interacts with mast cells causing mediator release, which may contribute to airway narrowing, obstruction, and wheezing. Welliver *et al*. [22] observed increased RSV-specific IgE in nasal secretions of wheezing children with acute RSV infection, but other groups have not been able to confirm that finding.

### Animal studies

The mouse model of RSV disease is well characterized and has given rise to a wealth of immunologic information regarding antiviral defense and immunopathogenic mechanisms [23]. Virus replication peaks on day 4 or 5 after mice are exposed to the pathogen, and cultivable virus is cleared between days 7 and 9. However, PCR for viral RNA is still positive for up to 100 days or more after infection. In terms of the cellular immune response, infected mice show few changes for the first 3 days, but on day 4 lymphocytes start to be recruited to the airway epithelium. Natural killer cells are initially abundant in bronchoalveolar lavage fluid and produce IFN-γ. Over the next few days these cells are replaced by Th1 CD4^+^ and CD8^+^ cells, which also produce predominantly IFN-γ during primary infection of unvaccinated animals.

**Figure 2:**
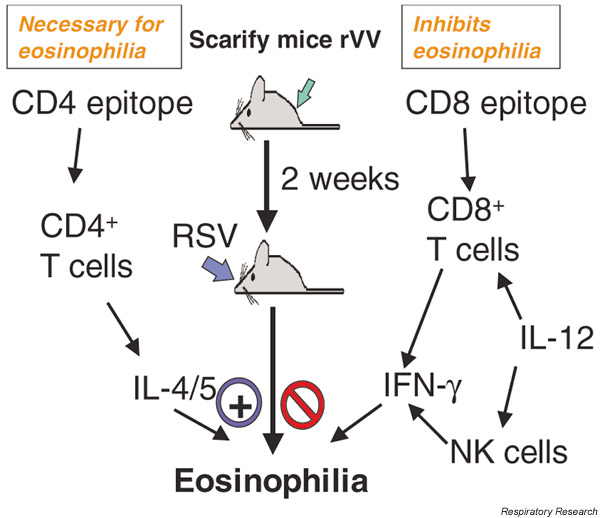
Production of lung eosinophilia in the mouse model of respiratory syncytial virus (RSV). Lung eosinophilia is linked to the inability of the major surface glycoprotein G of RSV (RSV G-protein) to stimulate T-helper (Th)1, or CD8^+^, cells to produce IFN-γ. Once activated, Th1 cells stimulate macrophages and dendritic cells to produce IL-12. This induces natural killer (NK) cells and Th1 cells to produce IFN-γ. In the presence of IFN-γ lung eosinophilia is inhibited; in its absence the Th2 response persists. Th2 cells produce IL-4 and IL-5, which causes lung eosinophilia. Adapted with permission from Openshaw *et al*. [25]. rVV, recombinant vaccinia virus.

The cytokines produced determine the predominant type of pathology observed [24]. Openshaw *et al*. [25] reported that early IFN-γ production is a key controlling element of Th1/2 balance during subsequent infection (Fig. 2). Without IFN-γ a Th2 cytokine profile persists, which leads to lung eosinophilia. Priming with the major RSV surface glycoprotein G expressed in a vaccinia virus vector (recombinant vaccinia virus [rVV]-G) fails to stimulate IFN-γ production by infiltrating cells during pulmonary challenge, leading to Th2 cytokine production and to lung eosinophilia. In mice primed with rVV expressing the fusion protein F, Th1 cells produce IFN-γ, inhibiting lung eosinophilia.

As stated above, in the absence of IFN-γ the Th2 response persists. Th2 cells produce IL-4 and IL-5; the latter is associated with delayed hypersensitivity and causes lung eosinophilia [26]. When this response occurs in infants, it permits the establishment of the Th2 response as part of T cell memory, which is long lasting.

The immunologic response may also be determined by genetic factors. In different strains of mice, RSV infected animals exhibited different Th1 or Th2 responses [27]. Hence, the pathogenesis of bronchiolitis inevitably varies between species and between individuals with different genetic and environmental backgrounds.

## Delayed effects of respiratory syncytial virus infection

A recent study found that T-cell activation (as measured by plasma levels of soluble serum IL-2 receptor, sCD25) was elevated for 5 months after infantile bronchiolitis, regardless of severity [28]; this is in marked contrast to the rapid normalization of soluble IL-2 receptor levels in other acute viral infections. Such studies suggest that effects from RSV infections may be responsible for respiratory problems later in life. Increased respiratory symptoms have been noted in children who have recovered from RSV bronchiolitis, and this association lasts for 8–13 years [29,30]. Sigurs *et al.*[31] followed 47 children treated for RSV bronchiolitis as infants for 3 years and subsequently for 7–8 years [5]. Compared with controls, the RSV-infected patients had a higher rate of asthma diagnosis (23% versus 1%) and reductions in measurements of airflow; no differences in lung size were observed. Noble *et al*. [32] reported similar findings in 61 patients followed for 9.5 years after hospital admission for bronchiolitis. Compared with controls, children who had been treated for bronchiolitis had a higher frequency of cough and wheeze, and increased bronchodilator use. In another study, persistent wheeze at the age of 7–8 years was directly related to the level of RSV-specific IgE measured in the nasopharyngeal secretions during the initial illness, which occurred during the first 6 months of life [33]. No change in lung function was reported at 7–8 years of age. Similarly, peripheral blood eosinophilia at the time of acute bronchiolitis was found to predict wheezing at 7 years of age [34].

From data obtained in mouse experiments, it was hypothesized that initial RSV infection increased Th2 sensitization to subsequent respiratory infections and to inhaled antigen. To test this hypothesis, IL-4 and IFN-γ production, and proliferative responses to RSV antigens and nonviral allergens was measured in peripheral blood cells of 7- and 8-year-old children [5]. RSV-specific enhancement of IL-4 production was found in children with a history of bronchiolitis, whereas all exposed children (regardless of the severity of first infection) showed good IFN-γ responses [35]. This finding is in agreement with studies by Teran *et al*. [36], who reported asthmatic exacerbations following viral infection altered cytokine responses.

Animal studies generally support the idea that there is a direct causal link between RSV infection and delayed wheezing disease. In guinea pigs, RSV infection causes increased sensitivity to inhaled histamine for at least 6 weeks [37]. Brown Norway rats develop chronic, episodic, and reversible airway obstruction after bronchiolitis [38]. This condition is produced by a Th2 response with reduced IFN-γ production. In cattle, viral persistence in B cells has been demonstrated [7].

If the initial RSV infection causes permanent damage to the epithelium, cilia, or lung structure, then it may be easier for antigens to penetrate the lung [6]. In addition, clearance of material from the lung may be impaired. These factors may leave the lung vulnerable to later colonization with a nonviral pathogen that could have delayed effects or cause secondary infection. Other possible mechanisms for the delayed effect are virus chronicity, persistence or latency [4,39], and immunologic tolerance [23].

**Figure 3:**
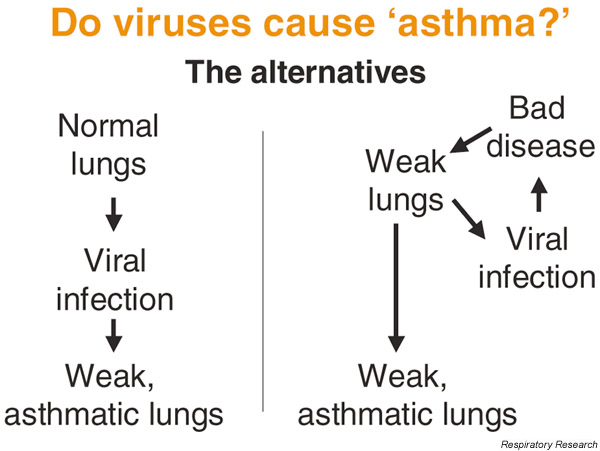
Delayed effects of acute respiratory syncytial virus (RSV) infection: possible mechanisms. The link between RSV infection and delayed respiratory problems may be explained by a severe initial infection, which either alters the host lungs or immunity, leading to future wheeze, or reveals an underlying tendency to wheeze. Adapted with permission from Balfour-Lynn and Openshaw [41].

Two hypotheses have been suggested [40,41] to explain this phenomenon (Fig. 3). The first is that early viral infections directly damage the developing lung or alter host immunity, and result in recurrent respiratory symptoms and subsequent airway dysfunction. The second hypothesis is that initial respiratory infection unmasks an inherent susceptibility to long-term respiratory problems. Both of these factors may contribute to delayed respiratory problems in some individuals, and are not mutually exclusive.

## Conclusion

If early RSV infection causes delayed respiratory problems, then prevention or delay of neonatal infection is highly desirable (Table 1). Delaying RSV infection would allow the lungs to mature and the immune system to develop and protect infants against sequelae caused by viral chronicity or persistence, a 'hitchhiking' pathogen, lung remodeling, immunologic memory, and bystander sensitization. Preventing RSV infections would not alter delayed effects of previously small lungs or genetic predisposition.

The long-term results of studies of passive or active immunization against RSV disease are therefore keenly awaited. New studies are underway that aim to determine how immune-specific responses can be directed to Th1 or Th2, in the hope that stimulating Th1 responses during the first exposure will be beneficial and reduce the long-term sequelae of natural RSV infection.

Progress is being made with vaccines based on viral subunits, live attenuated strains of RSV, DNA vaccines, and synthetic peptides [42-44]. In ongoing clinical trials, vaccination of breast-feeding women appears to protect neonates from RSV infection during the critical first few months of life [45]. The results indicate the presence of RSV-specific antibody in breast milk is not associated with adverse effects on neonatal or infant immune responses to RSV, but more studies are underway.

## Abbreviations

IFN = interferon; IL = interleukin; MIP = macrophage inflammatory protein; RANTES = regulated on activation, normal T cell expressed and secreted; RSV = respiratory syncytial virus; rVV = recombinant vaccinia virus; Th = T-helper; TNF = tumor necrosis factor.

## Figures and Tables

**Table 1:** Challenges for effective treatment and vaccination of respiratory syncytial virus

**Aim**
Prevent RSV bronchiolitis in infants under 6 months:
Maternal of neonatal vaccination
Passive antibody administration
**Studies are required:**
To identify factors that induce Th1 versus Th2 responses
To gain an understanding of tolerance and immune suppression
To gain an understanding the role of nonprotein antigens
To control IgE responses
To boost IgA responses
To make practical improvements to DNA vaccination
To develop new adjuvants

RSV, respiratory syncytial virus; Th, T-helper.

## References

[B1] Shay DK, Holman RC, Newman RD, Liu LL, Stout JW, Anderson LJ (1999). Bronchiolitis-associated hospitalizations among US children, 1980–1996.. JAMA.

[B2] Anderson LJ, Heilman CA (1995). Protective and disease-enhancing immune responses to respiratory syncytial virus.. J Infect Dis.

[B3] Parrott RH, Kim HW, Arrobio JO, Hodes DS, Murphy BR, Brandt CD, Camargo E, Chanock RM (1973). Epidemiology of respiratory syncytial virus infection in Washington, D.C.: II. Infection and disease with respect to age, immunologic status, race and sex.. Am J Epidemiol.

[B4] Openshaw PJ, Hewitt C (2000). Protective and harmful effects of viral infections in childhood on wheezing disorders and asthma.. Am J Respir Crit Care Med.

[B5] Sigurs N, Bjarnason R, Sigurbergsson F, Kjellman B (2000). Respiratory syncytial virus bronchiolitis in infancy is an important risk factor for asthma and allergy at age 7.. Am J Respir Crit Care Med.

[B6] O'Donnell DR, Openshaw PJ (1998). Anaphylactic sensitization to aeroantigen during respiratory virus infection.. Clin Exp Allergy.

[B7] Valarcher JF, Bourhy H, Lavenu A, Bourges-Abella N, Roth M, Andreoletti O, Ave P, Schelcher F (2001). Persistent infection of B lymphocytes by bovine respiratory syncytial virus.. Virology.

[B8] Noah TL, Henderson FW, Wortman IA, Devlin RB, Handy J, Koren HS, Becker S (1995). Nasal cytokine production in viral acute upper respiratory infection of childhood.. J Infect Dis.

[B9] Einarsson O, Geba GP, Zhu Z, Landry M, Elias JA (1996). Interleukin-11: stimulation in vivo and in vitro by respiratory viruses and induction of airways hyperresponsiveness.. J Clin Invest.

[B10] Calhoun WJ, Swenson CA, Dick EC, Schwartz LB, Lemanske RF, Busse WW (1991). Experimental rhinovirus 16 infection potentiates histamine release after antigen bronchoprovocation in allergic subjects.. Am Rev Respir Dis.

[B11] Everard ML, Swarbrick A, Wrightham M, McIntyre J, Dunkley C, James PD, Sewell HF, Milner AD (1994). Analysis of cells obtained by bronchial lavage of infants with respiratory syncytial virus infection.. Arch Dis Child.

[B12] Teran LM, Seminario MC, Shute JK, Papi A, Compton SJ, Low JL, Gleich GJ, Johnston SL (1999). RANTES, macrophage-inhibitory protein 1alpha, and the eosinophil product major basic protein are released into upper respiratory secretions during virus-induced asthma exacerbations in children.. J Infect Dis.

[B13] Harrison AM, Bonville CA, Rosenberg HF, Domachowske JB (1999). Respiratory syncytial virus-induced chemokine expression in the lower airways: eosinophil recruitment and degranulation.. Am J Respir Crit Care Med.

[B14] Isaacs D, Bangham CRM, McMichael AJ (1987). Cell-mediated cytotoxic response to respiratory syncytial virus in infants with bronchiolitis.. Lancet.

[B15] Welliver RC (1999). Immunologic mechanisms of virus-induced wheezing and asthma.. J Pediatr.

[B16] Romagnani S (1992). Induction of TH1 and TH2 responses: a key role for the 'natural' immune response?. Immunol Today.

[B17] Folkerts G, Busse WW, Nijkamp FP, Sorkness R, Gern JE (1998). Virus-induced airway hyperresponsiveness and asthma.. Am J Respir Crit Care Med.

[B18] Openshaw PJM, Pala P, Sparer T, Matthews S, Pennycook A, Hussell T (2000). T-cell subsets and lung inflammation: lessons from respiratory syncytial virus.. Eur Respir Rev.

[B19] Glezen WP, Paredes A, Allison JE, Taber LH, Frank AL (1981). Risk of respiratory syncytial virus infection for infants from low-income families in relationship to age, sex, ethnic group, and maternal antibody level.. J Pediatr.

[B20] Kaul TN, Faden H, Ogra PL (1981). Effect of respiratory syncytial virus and virus-antibody complexes on the oxidative metabolism of human neutrophils.. Infect Immun.

[B21] Nadal D, Ogra PL (1990). Development of local immunity: role in mechanisms of protection against or pathogenesis of respiratory syncytial viral infections.. Lung.

[B22] Welliver RC, Kaul TN, Ogra PL (1980). The appearance of cell-bound IgE in respiratory-tract epithelium after respiratory-syncytial-virus infection.. N Engl J Med.

[B23] Openshaw PJ (2001). Potential mechanisms causing delayed effects of respiratory syncytial virus infection.. Am J Respir Crit Care Med.

[B24] Lemanske RF, Hiatt PW (1998). Immunologic mechanisms in RSV-related allergy and asthma.. In RSV and Asthma: Is There a Link?.

[B25] Openshaw PJM, Matthews S, Pala P, Hussell T, Walzl G, Wardlaw AJ, Hamid QA (2002). Immunopathogenesis of viral infections in children.. In Textbook of Respiratory Cell and Molecular Biology.

[B26] Alwan WH, Kozlowska WJ, Openshaw PJM (1994). Distinct types of lung disease caused by functional subsets of antiviral T cells.. J Exp Med.

[B27] Hussell T, Georgiou A, Sparer TE, Matthews S, Pala P, Openshaw PJM (1998). Host genetic determinants of vaccine-induced eosinophilia during respiratory syncytial virus infection.. J Immunol.

[B28] Smyth RL, Fletcher JN, Thomas HM, Hart CA, Openshaw PJM (1999). Respiratory syncytial virus and wheeze.. Lancet.

[B29] McConnochie KM, Roghmann KJ (1989). Wheezing at 8 and 13 years: changing importance of bronchiolitis and passive smoking.. Pediatr Pulmonol.

[B30] Stein RT, Sherrill D, Morgan WJ, Holberg CJ, Halonen M, Taussig LM, Wright AL, Martinez FD (1999). Respiratory syncytial virus in early life and risk of wheeze and allergy by age 13 years.. Lancet.

[B31] Sigurs N, Bjarnason R, Sigurbergsson F, Kjellman B, Bjorksten B (1995). Asthma and immunoglobulin E antibodies after respiratory syncytial virus bronchiolitis: a prospective cohort study with matched controls.. Pediatrics.

[B32] Noble V, Murray M, Webb MSC, Alexander J, Swarbrick AS, Milner AD (1997). Respiratory status and allergy nine to 10 years after acute bronchiolitis.. Arch Dis Child.

[B33] Welliver RC, Duffy L (1993). The relationship of RSV-specific immunoglobulin E antibody responses in infancy, recurrent wheezing, and pulmonary function at age 7–8 years.. Pediatr Pulmonol.

[B34] Ehlenfield DR, Cameron K, Welliver RC (2000). Eosinophilia at the time of respiratory syncytial virus bronchiolitis predicts childhood reactive airway disease.. Pediatrics.

[B35] Pala P, Bjarnason R, Sigurbergsson F, Metcalfe C, Sigurs N, Openshaw PJM (2002). Enhanced IL-4 responses in children with a history of respiratory syncytial virus bronchiolitis in infancy.. Eur Respir J.

[B36] Teran LM, Johnston SL, Schroder JM, Church MK, Holgate ST (1997). Role of nasal interleukin-8 in neutrophil recruitment and activation in children with virus-induced asthma.. Am J Respir Crit Care Med.

[B37] Robinson PJ, Hegele RG, Schellenberg RR (1997). Allergic sensitization increases airway reactivity in guinea pigs with respiratory syncytial virus bronchiolitis.. J Allergy Clin Immunol.

[B38] Kumar A, Sorkness RL, Kaplan MR, Lemanske RF (1997). Chronic, episodic, reversible airway obstruction after viral bronchiolitis in rats.. Am J Respir Crit Care Med.

[B39] Dakhama A, Vitalis TZ, Hegele RG (1997). Persistence of respiratory syncytial virus (RSV) infection and development of RSV-specific IgG1 response in a guinea-pig model of acute bronchiolitis.. Eur Respir J.

[B40] Long CE, McBride JT, Hall CB (1995). Sequelae of respiratory syncytial virus infections. A role for intervention studies.. Am J Respir Crit Care Med.

[B41] Balfour-Lynn IM, Openshaw PJM, Silverman M (2002). Viral infection.. In Childhood Asthma and Other Wheezing Disorders.

[B42] Dudas RA, Karron RA (1998). Respiratory syncytial virus vaccines.. Clin Microbiol Rev.

[B43] Crowe JE (1998). Immune responses of infants to infection with respiratory viruses and live attenuated respiratory virus candidate vaccines.. Vaccine.

[B44] Olszewska W, Zambon M, Openshaw PJM (2002). Vaccination against common colds.. Br Med Bull.

[B45] Englund J, Glezen WP, Piedra PA (1998). Maternal immunization against viral disease.. Vaccine.

